# Our First Hospital Saturday

**Published:** 1888-07-07

**Authors:** A. W. Webster

**Affiliations:** Chairman of Workmen's Governors, Sunderland Infirmary.


### Our First Hospital Saturday.

By A. W. Webster, Chairman of Workmen's Governors, Sunderland Infirmary.

Who can ever mistake a picture of "John Bull"? Is he not a portly, full-blooded gentleman, giving the impression that the application of a horse-leech to his veins for an hour would produce nothing worse than increased agility? Is he not comfortably and neatly clad, and do not his slight but costly adornments indicate happy relations with his banker? The typical "John" is the very beau-ideal of a squeezable gentleman, but should the operation of squeezing not be judiciously and skilfully performed, woe betide the squeezer. "John," of course, is a dignified gentleman, as becomes his nationality. He is proud of his country's greatness, and boasts of her free institutions. "Look," says he, "at the thousands of hospitals, convalescent homes, orphanages, training schools, and kindred institutions supported by the voluntary contributions of my countrymen." Ah, John! I wonder what proportion of your subscriptions finds its way to these establishments without the operation of squeezing? Support the hospitals? Of course you will. But, then, what a dreadful lot of manœuvring and planning it takes to reach the bottom of your breeches pocket. True, your name may be found on the list of guinea subscribers, but then it should be five or ten guineas instead of one; and however little the knowledge of this fact may disturb your placid equilibrium, the ingenuity of the hospital manager must be constantly at work, devising means to cajole another guinea or two from your well-filled coffers.

Necessity is said to be the mother of invention, and our hospital authorities have long ago discovered the truth of this proverb. Endowments, legacies, and all the annual conventional guinea subscriptions combined are totally inadequate to meet the demand, and managers must face the two alternatives—bankruptcy, or the application of the squeezer. Foremost among the extraneous efforts to assist in the support of our medical charities is the Hospital Sunday movement, and considering that it has barely reached its majority, its growth in all parts of the country may be looked upon as rapid, and creditable alike to its founders and supporters. A few years' experience soon convinced the promoters of the utility of their scheme, and the success of appealing to a church-going community seems to have inspired the idea that a similar appeal could be made to workmen by establishing a Hospital Saturday. Although this latter movement was founded in London fifteen years ago, its growth has not been so marked as in some provincial towns where its introduction dates from more recent times. An appeal once a year to workmen in receipt of small weekly wages has never produced large results, either in London or elsewhere, and the original idea soon gave way to the plan of more frequent appeals by the aid of collecting sheets and the permanent fixing of boxes in the workshops. Street collections on Hospital Saturday were also added to this movement, and the revenue from this source is now considerable. Hospital Saturday as a fixed day once a year for receiving subscriptions from workmen may be considered a failure, and the sooner a weekly system of contribution is substituted the better for all concerned. Get your workmen's pennies week by week, and remodel your Hospital Saturday so as to efficiently appeal to the entire community—this is the kernel of my present suggestion. It is well known that workmen's contributions flow into many of our provincial hospitals, where a Hospital Saturday is unknown. Such places as these may also take the hint, and bestir themselves to establish a movement, which is sure to prove a permanent source of income to their respective institutions. Our hospital in Sunderland is about equally supported by the rich and poor —that is, one-half of the revenue is supplied by the well-to do citizens, and the other half by the workpeople. There was no need of a Hospital Saturday to reach our workmen, but there was great need of one to reach the class above them, who gave little or nothing to the support of their charities.

How to reach this class in particular, and the unsubscribing members of all other classes, was the problem, and our workmen's (hospital) organisation solved the difficulty completely by establishing a Hospital Saturday, the main feature of which was a house-to-house visitation for subscriptions. A superintendent and a few assistants were appointed for each municipal ward in the borough, and between the hours of one and eight o'clock a thorough systematic canvas was made, each collector being armed with a sealed box into which the public were warned to put all contributions. Boxes were also placed in hotels and cafés, and a few in the streets. The experiment can be pronounced a complete success, and worthy of imitation in othes places. Our collectors, many of whom were previously dubious about the public toleration of itinerant beggars, were delighted with the reception given them by all classes, and "John Bull" fully maintained his reputation of a squeezable gentleman. Very few indeed resented the operation. Where this occurred perhaps the squeezer was at fault. One collector came in contact with a relation of Goldscraper's, in Moneygrabbing Crescent. The silent appeal of a legible label on the Hospital Saturday box had no effect on his hard heart. A blunt request got no nearer Goldscraper's well-lined purse. Rehearsing the worthiness of the object produced a scowl. Details of the efficient and economic management of the institution brought forth a lecture on political economy as long as my arm. A reminder that the poor in our town helped themselves far more than in others, gave Goldscraper such a splendid opportunity for expatiating on the principles and motives which governed his actions, that our collector was compelled to beat a hasty retreat in the midst of a beautiful and effective peroration. Confusion reigned for half an hour after. Goldscraper had muddled our collector's head, but had not deigned to touch his box. A street was missed, and the mistake was not discovered till Monday morning, when no collecting could be done. Our chagrin was lessened when resident Number One stepped into our secretary's office saying, "Our street was not canvassed on Saturday, but here is seventeen and sixpence from our family and Saturday visitors which awaited your collector." Shortly after resident Number Five called in with half a-crown.

Listen, Mr. Goldscraper. The sight of one of our boxes settled a most wordy warfare between two fishwives in half a minute, and one of the combatants forked out her last penny in honour of the occasion. Another collector called at a house, where he was requested by a girl of twelve or thirteen to come to the bedside of her mother—a poor bedridden invalid—to receive a contribution. A scantily-furnished house showed few signs of plenty, and certainly none of abundance; yet this poor sufferer deposited seven shillings in the box, and gave it from a full heart, and with a blessing. An ex-patient of the local hospital was unearthed about the same time living in a miserable hovel. Not a penny in the world had she, but something must be given to the institution from which she had derived so much benefit. Would the collector wait a few minutes, or go up the next stair till she returned? Yes, he would do either, though he was only asking from those who were able to give. Five minutes elapsed when our ex-patient returned minus a garment, which she had pledged to enable her to contribute sixpence to our Hospital Saturday Collection. One more typical incident and I have done. An enthusiastic collector was energetically working a street—two doors at a time—when a shrill but somewhat feeble cry of "Echo and Post" announced the near approach of a young female newsvendor. The girl was sparingly clad, and had all the appearance of belonging to a very poor family. One paper had been sold, and immediately the little maiden recognised the Hospital Saturday box in the hands of our collector, permission was asked to contribute her solitary halfpenny to the infirmary. "Echo and Post" again rang forth in shrill, clear tones; a purchaser was found, a second journey made to our collector's box, and in dropped halfpenny number two. Three, four, five journeys did this young angel of mercy make to the box, each time panting for breath, but with a countenance beaming with joy and satisfaction that bespoke the earnestness of the spirit within.

Thus was ushered into the world our first Hospital Saturday, born on June 9th, 1888, under auspicious circumstances, strong in its infancy, and bright with promise for the future.

